# Sphingosine-1-Phosphate Signaling in Ischemic Stroke: From Bench to Bedside and Beyond

**DOI:** 10.3389/fncel.2021.781098

**Published:** 2021-11-30

**Authors:** Shuo-Qi Zhang, Jun Xiao, Man Chen, Luo-Qi Zhou, Ke Shang, Chuan Qin, Dai-Shi Tian

**Affiliations:** ^1^Department of Neurology, Tongji Hospital, Tongji Medical College, Huazhong University of Science and Technology, Wuhan, China; ^2^Department of Radiology, Tongji Hospital, Tongji Medical College, Huazhong University of Science and Technology, Wuhan, China

**Keywords:** sphingosine-1-phosphate, ischemic stroke, fingolimod, neuroprotective agent, translational research

## Abstract

Sphingosine-1-phosphate (S1P) signaling is being increasingly recognized as a strong modulator of immune cell migration and endothelial function. Fingolimod and other S1P modulators in ischemic stroke treatment have shown promise in emerging experimental models and small-scale clinical trials. In this article, we will review the current knowledge of the role of S1P signaling in brain ischemia from the aspects of inflammation and immune interventions, sustaining endothelial functions, regulation of blood-brain barrier integrity, and functional recovery. We will then discuss the current and future therapeutic perspectives of targeting S1P for the treatment of ischemic stroke. Mechanism studies would help to bridge the gap between preclinical studies and clinical practice. Future success of bench-to-bedside translation shall be based on in depth understanding of S1P signaling during stroke and on the ability to have a fine temporal and spatial regulation of the signal pathway.

## Introduction

As the second largest cause of death and a leading cause of disability (Johnson et al., 2019), stroke produces immense health and economic burdens globally (Virani et al., 2020). With an aging population, the prevalence of stroke can be predicted to increase. More worrisome is the fact that the incidence of stroke in young adults has increased in recent years, which causes a profound socioeconomic impact due to high health-care expenditure and compromised labor productivity (Ekker et al., 2018). Most stroke survivors are unable to live independently and have greater risks of recurrence and other long-term disabling sequelae such as dementia (Levine et al., 2015).

Although the primary health goal is a decrease in stroke incidence as prevention is always better than cure. The increasing global stroke burden indicates that primary prevention strategies may not be sufficiently effective (Johnson et al., 2019), which calls for effective therapies in the acute phase and long-term follow-up rehabilitation for people who have developed stroke. About 87% of all strokes are ischemic (Virani et al., 2020). Despite a series of clinical trials with neuroprotective drugs, current treatment options remain limited to thrombolysis and mechanical recanalization for the acute phase of ischemic stroke (Powers, 2019). While effective, the treatments can only be applied to less than 10% of patients for a narrow treatment time window and strict exclusion criteria (Rinaldo et al., 2019). Moreover, even a successful recanalization may lead to enlarged infarction due to ischemic-reperfusion (IR) injury (Mizuma et al., 2018). Novel safe and effective treatment strategies are therefore needed.

**FIGURE 1:**
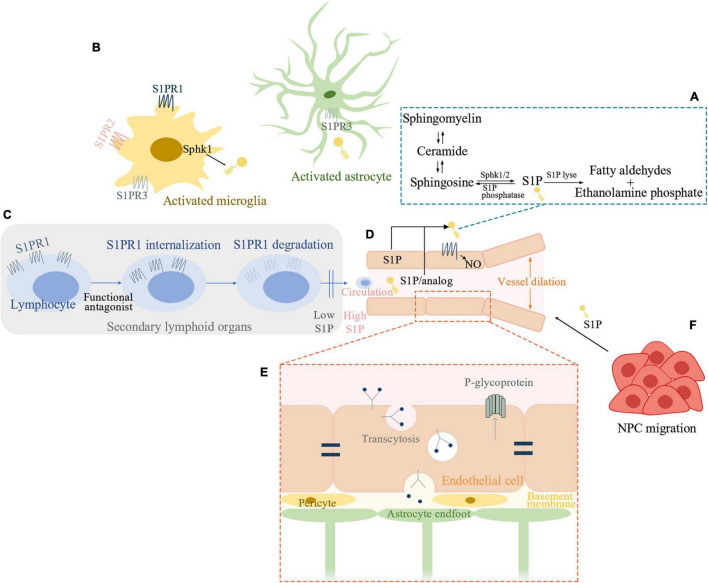
Sphingosine1-phosphate (S1P) signaling pathway involved in ischemic stroke. **(A)** Schematic outline of S1P metabolism. **(B)** S1P signaling in post-ischemic brain inflammation. Sphk1 level is elevated in microglia and S1P production is enhanced in activated microglia. S1PR1, S1PR2, and S1PR3 influence microglial activation. Astrocytes interact extensively with microglia. S1P can activate astrocytes while S1PR3 is known to promote astrogliosis in ischemic brain. **(C)** S1P and recruitment of peripheral lymphocytes. Functional antagonist of S1PR1 can induce receptor internalization and degradation and thereby inhibits peripheral lymphocytes’ recruitment. **(D)** S1P signaling in sustaining endothelial functions. S1P-S1PR1-nitric oxide regulates vessel dilation to flow. S1PR1 distributes to the abluminal surface of the endothelial cells. Either cell-autonomous S1P or system S1P/analog which penetrates the blood-brain barrier (BBB) can activate endothelial S1PR1 and sustains endothelial function. **(E)** S1P inhibits the function of P-glycoprotein at the BBB. Brain endothelial S1PR1 may help maintain BBB function by sustaining a proper distribution of tight junction proteins. Endothelial S1PR1 may participate in regulating vesicular transport in the early phase of BBB opening after ischemic stroke. **(F)** S1P is a chemoattractant for neural progenitor cells (NPC) toward the infarcted area thus facilitating neurogenesis. Sphk, sphingosine kinase; S1P, sphingosine-1-phosphate; S1PR, S1P receptor; NO, nitric oxide; NPC, neural progenitor cells.

Sphingosine-1-phosphate (S1P) is a terminal breakdown product of sphingolipid metabolism discovered in the 1960s (Cartier and Hla, 2019). The degradation of plasma membrane sphingomyelin produces ceramide (Merrill, 2011). S1P is then produced through the metabolism of ceramide by ceramidase and two types of sphingosine kinases (SphK1 and −2). S1P can also be recycled back to ceramide or irreversibly degraded by S1P lyse (Figure 1; Hannun and Obeid, 2018). Many tissues keep a low intracellular S1P level by a rapid degradation of S1P. By contrast, S1P can be transported extracellularly allowing its extracellular action (Proia and Hla, 2015). Erythrocytes and endothelial cells are the major producers of S1P, while substantial amounts of S1P stored in platelets can only be released upon activation (Gazit et al., 2016). In circulation, S1P is bound mainly to high-density lipoproteins and albumin (Proia and Hla, 2015). These protein chaperones of S1P enable its solubility and specific biological activity. Outside the cell, S1P binds to S1P receptors (S1PR) to exert its biological actions. Vertebrates possess five S1PR (S1PR1-5) which are G-protein coupled receptors (Proia and Hla, 2015). These widely expressed S1PRs couple to key intracellular signaling pathways (Chun et al., 2010; Proia and Hla, 2015), thus coupling phospholipid metabolism with intercellular communication (Cartier and Hla, 2019). In the central nervous system, neuronal lineages express all S1PR (Soliven et al., 2011). Astrocytes and microglia express predominantly S1PR1 and S1PR3 (Benarroch, 2021). S1PR5 is mainly expressed in oligodendrocytes (di Nuzzo et al., 2014). As for peripheral immune cells, T cells are known to express S1PR1 (Baeyens et al., 2021), S1PR2 (Baeyens et al., 2015), and S1PR4 (Xiong et al., 2019). Human B cells express S1PR1, S1PR2, and S1PR4 at different levels by different B-cell subtypes but not S1PR3 (Sic et al., 2014). S1P signaling participates in many processes of growth and development and pathological conditions (Proia and Hla, 2015). It is essential for neural and vascular development (Mizugishi et al., 2005). S1P is in relatively high concentrations in circulation compared with tissue parenchyma. This concentration gradient, formed by the interplay of S1P synthetic and degradative enzymes as well as S1P exporters, is fundamental to S1P biology such as regulating lymphocyte migration (Cyster and Schwab, 2012) and supporting endothelial barrier function (Camerer et al., 2009).

The observation that *Rag1^–/–^* mice, that were mice devoid of lymphocytes, developed smaller infarct volume than that of wild-type mice (Yilmaz et al., 2006), pioneering a new field of immunomodulating therapies in ischemic stroke, especially those that have been used for the treatment of multiple sclerosis (MS) (Dreikorn et al., 2018). One of the best-studied drugs is fingolimod (FTY720), a S1P modulator that causes rapid induction of lymphopenia (Cyster and Schwab, 2012) and is approved for the treatment of relapsing-remitting multiple sclerosis (RRMS). Moreover, recent studies suggest that the function of S1P signaling in ischemic stroke includes but goes far beyond immunomodulating (Li et al., 2020; Chua et al., 2021; Nitzsche et al., 2021). Emerging experimental models and small-scale clinical trials have shown promise of S1P modulator for the treatment of stroke (Cyster and Schwab, 2012; Kraft et al., 2013; Fu et al., 2014; Qin et al., 2017). Herein, we highlight S1P signaling pathway in ischemic stroke and the translation from biomedical research basis into clinical stroke applications.

## S1P in Cerebral Ischemia

We attempt to have a brief summary from the aspects of brain inflammation and immune interventions, sustaining endothelial functions, regulation of blood-brain barrier (BBB), and functional recovery (Figure 1).

### S1P Signaling in Post-ischemic Brain Inflammation and Immune Interventions

Inflammatory reactions and immune responses have long been recognized as important elements during ischemic stroke (Fu et al., 2015). Neural cell death after ischemia releases damage-associated molecular patterns (DAMPs) and triggers immune responses including activation of microglia and astrocytes, recruitment of resident and peripheral immune cells (Shi et al., 2019). Inflammation can be both detrimental and beneficial at certain stages after stroke (Lambertsen et al., 2018).

Microglia are among the very first cell types to be activated and recruited to the site of ischemia (Ma et al., 2017). Upon activation, microglia produce numerous mediators such as cytokines and chemokines, growth and trophic factors (Ma et al., 2017). Studies showed that Sphk1 level was elevated in microglia and S1P production was enhanced mainly in activated microglia after ischemia (Kimura et al., 2008; Zheng et al., 2015). The production of S1P on the other hand enhanced the release of proinflammatory mediators from microglia (Nayak et al., 2010; Moon et al., 2015). In addition, S1PR1, S1PR2, and S1PR3 were shown to influence microglial activation and inflammation (Gaire et al., 2018b,^2019^; Sapkota et al., 2019; Gaire and Choi, 2021). S1PR1 knock-down reduced microglial activation and microglial proliferation after ischemia (Gaire et al., 2018a). Therefore, the pathogenic role of S1P signaling may have a close relationship with microglia activation after ischemic stroke.

As the most numerous cells in the brain, astrocytes interact extensively with microglia (Liddelow et al., 2020) and help recruit immune cells (Li M. et al., 2017). S1P was also shown to activate astrocytes (Sorensen et al., 2003) while S1PR3 could promote astrogliosis after ischemic stroke (Gaire et al., 2018a,b). An antagonist of S1PR3, CAY10444 was found to attenuate astrocyte activation after transient middle cerebral artery occlusion (tMCAO) (Gaire et al., 2018b). Furthermore, S1PR3 deletion could attenuate S1P-induced inflammatory responses in astrocytes (Dusaban et al., 2017).

Invasion of peripheral lymphocytes drives the progression of inflammation (Shi et al., 2019). During IR injury, T cells interact with platelets and formulate a complex process called thrombo-inflammation which leads to infarct expansion (Stoll and Nieswandt, 2019). Experiments on a series of ischemic stroke mouse models indicated that the protective effect of S1P modulators such as fingolimod could be largely attributed to impairment of lymphocyte trafficking and thereby, lymphocyte-driven thrombo-inflammation (Kraft et al., 2013). A marked decrease in infarct volume and improvement of functional outcome were found after fingolimod administration in wild type but not lymphocyte-deficient *Rag1^–/–^* mice after tMCAO, highlighting the key mechanism of lymphocytopenia in its protective effect (Kraft et al., 2013). Lymphocytopenia could then attenuate thrombo-inflammation in microvasculature and increase cerebral blood flow (CBF) after tMCAO (Kraft et al., 2013). However, in another permanent occlusion of the middle cerebral artery (pMCAO) mouse model, infarct volume and behavioral dysfunction were not reduced by fingolimod (Liesz et al., 2011). Similar results were found in pan-hematopoietic *S1pr1* knockout (KO) mice. Hematopoietic S1PR1 deficiency induced lymphopenia and exerted some neuroprotection after tMCAO, but not pMCAO (Nitzsche et al., 2021). These unexpected negative findings in pMCAO can be explained by the difference in the contribution of neuroinflammation in transient and permanent ischemia (Stoll and Nieswandt, 2019). In contrast to tMCAO where recanalization occurs, and the function of T cells is well established as contributing to IR injury in an antigen-independent fashion, in pMCAO, the contribution of T cells is less clear and more complex as secondary phenomena such as gut microbiome mediated systemic immunomodulation and stroke-related immunodepression syndrome may participate in the process (Stoll and Nieswandt, 2019).

### S1P Signaling in Sustaining Endothelial Functions

Cerebral endothelial dysfunction contributes to stroke-induced brain injury. Despite successful recanalization, as many as 25–50% of patients still have undesirable long-term outcomes (Meinel et al., 2020). A main reason for this futile recanalization is poor reperfusion in the microvasculature downstream of an occlusion (Tian et al., 2018). In addition, to counteract the expansion of the infarct core, adequate and timely perfusion is needed to rescue the ischemic penumbra, the area surrounding the necrotic core, where CBF is still sufficient to keep neurons alive (Manning et al., 2014). Not only the number and size but also the dilatory capacity, integrity, and patency of collateral anastomose are important for successful reperfusion (Bonnin et al., 2019). Ischemia impairs endothelium-dependent vasodilation (Hu et al., 2017) and induces a proinflammatory endothelial phenotype (Ishikawa et al., 2003) which may promote thrombus formation and reduce blood flow. Sustaining endothelial functions in ischemic stroke may therefore constitute a therapeutic opportunity (Shuaib et al., 2011).

S1PR1, S1PR2, and S1PR3 are found in endothelial cells and S1P signaling help sustain endothelial functions (Proia and Hla, 2015). S1P is believed to act on endothelial S1PR1 to reduce vascular leakage (Camerer et al., 2009) and improve endothelial barrier (Huwiler and Zangemeister-Wittke, 2018). S1P-S1PR1-nitric oxide signaling was found to be a new regulatory pathway of vessel dilation to flow (Cantalupo et al., 2017). As for endothelial S1PR1 in the brain, a recent study demonstrated that after the formation of BBB, S1PR1 distributed to the abluminal surface of the endothelial cells which shielded them from ligands in circulation (Nitzsche et al., 2021). Therefore, cell-autonomous S1P is required to activate these endothelial S1PR1. While BBB penetration is needed for synthetic ligands to reach these receptors (Nitzsche et al., 2021). S1PR1 maintained endothelial function during cerebral ischemia as endothelial cell *S1pr1* knockout (*S1pr1^ECKO^*) mice showed impaired microvascular perfusion after pMCAO (Nitzsche et al., 2021). Moreover, failure of collateral formation was found in *S1pr1^ECKO^* mice after pMCAO (Nitzsche et al., 2021). Based on these findings, the study showed that a S1PR1-selective agonist which can readily penetrate BBB targeting at endothelial S1PR1 receptor pool provided protection against ischemic injury (Nitzsche et al., 2021). And this beneficial effect was independent of reperfusion, which was in contrast to that of lymphocytopenia.

### S1P Regulation of BBB Integrity

Lying between peripheral circulation and the brain parenchyma, BBB serves as both structural and metabolic barriers that restrict the access of many compounds while keeping the transport of nutrients and oxygen to the brain, thus maintaining the extracellular environment (Profaci et al., 2020). BBB permeability is controlled by cerebral endothelial cells along with pericytes and astrocytes through the presence of endothelial tight junctions (TJ), efflux and solute transporters, and low levels of transcytosis (Obermeier et al., 2013). During reperfusion, oxidative stress-induced BBB disruption causes vascular leakage which leads to vasogenic edema and aggravated brain damage. Moreover, extreme barrier disruption will result in intracerebral hemorrhage (Jickling et al., 2014). In experimental stroke studies, BBB opening is biphasic. A partial recovery was found between the initial and second increase in BBB permeability (Pillai et al., 2009). Two different mechanisms participated in this process, beginning with upregulation of endothelial transcytosis (6 h after tMCAO). Dynamic remodeling of TJ complexes forming gaps or protrusions is not obvious until in the late phase (24–48 h after tMCAO) (Knowland et al., 2014).

S1P has been reported to inhibit the function of P-glycoprotein at the BBB (Cannon et al., 2012). Since P-glycoprotein is an efflux pump for small-molecule drugs, S1P might therefore enhance drug transportation across the BBB (Cannon et al., 2012). *S1pr1^ECKO^* mice showed a size-selective BBB disruption and different subcellular distribution of tight junctional proteins in brain microvessels (Yanagida et al., 2017). Thus, brain endothelial S1PR1 may help maintain BBB function by sustaining a proper distribution of tight junction proteins. During ischemia, endothelial cell S1P signaling also plays important roles. Profound edema can be seen in *S1pr1^ECKO^* mice shortly after tMCAO by MRI (Nitzsche et al., 2021), which suggests that endothelial S1PR1 may participate in regulating vesicular transport in the early phase of BBB opening. In line with this, in brain arterioles, ApoM-S1P regulates vesicular transport through S1PR1 signaling (Janiurek et al., 2019). In contrast, S1PR2 was shown to induce cerebrovascular permeability in tMCAO mice by experiments using genetic approaches and a S1PR2 antagonist (Kim et al., 2015).

### S1P Signaling in Functional Recovery Following an Ischemic Stroke

A series of events such as neurogenesis, synaptogenesis, angiogenesis, and white matter remodeling (Sommer and Schabitz, 2021) happen after ischemic stroke that contribute to neural repair and functional recovery (Overman and Carmichael, 2014). S1P signaling could also be involved in these events. The migration of neural progenitor cells (NPC) is an important step in neurogenesis (Koh and Park, 2017). S1P has been shown as a chemoattractant for NPCs released from infarction zone and S1PR2 antagonism enhanced the migration of NPCs toward the infarcted area thus facilitating neurogenesis (Kimura et al., 2008). Besides, fingolimod was found to enhance angiogenesis in a photothrombotic stroke model (Shang et al., 2020). In another photothrombotic stroke model, fingolimod significantly decreased astrogliosis and increased post-synaptic densities up to a month after the onset of ischemia (Brunkhorst et al., 2013). Other roles of S1P signaling in functional recovery following an ischemic stroke are poorly understood and shall constitute a promising direction for future studies.

The contributions of S1P signaling in ischemic stroke are primarily through diverse mechanisms as mentioned above. Additional mechanisms warrant further exploration. Some researches supported a possible direct action of S1P signaling on neuronal function after ischemic stroke (Hasegawa et al., 2010, 2013), while others proposed that such influence might be limited. As fingolimod, an analog of sphingosine, penetrated the blood-brain barrier, but it was not primarily located in neurons (Miron et al., 2008). Besides, in neuronal cell cultures under hypoxic conditions, fingolimod did not reduce cell death (Kraft et al., 2013). With more studies being undertaken, joint targeting of diverse mechanisms is suggested.

## Insights Into Current and Future Therapeutic Perspectives

### Fingolimod

Fingolimod (Gilenya™, Novartis), is an analog of sphingosine and was the first oral treatment approved by the United States Food and Drug Administration for RRMS (Brinkmann et al., 2010). Fingolimod is phosphorylated by Sphk2 to fingolimod-phosphate (fingolimod-P) (Zheng et al., 2015), which is an agonist for four S1PR (S1PR1, 3, 4, and 5) (Brinkmann et al., 2002). Fingolimod-P activates S1P receptors with very high potency and efficacy. The over-activation leads to rapid receptor internalization and degradation, especially for S1PR1 (Cyster and Schwab, 2012). Thus, fingolimod serves as a functional antagonist of S1PR1. While S1PR3, 4, 5 are also internalized but can come back to the cell surface without being degraded (Huwiler and Zangemeister-Wittke, 2018).

The mechanism of action of fingolimod in ischemic stroke is mainly mediated by the functional antagonism and agonism of S1P receptors (Wang et al., 2020). Fingolimod binds to S1PR1 on lymphocytes and leads to transient receptor degradation, thereby preventing lymphocytes from releasing to the bloodstream (Brinkmann, 2007). As discussed above, this decreases post-stroke lymphocyte infiltration and therefore thrombo-inflammation formation and inflammatory response. Besides its role in immunomodulation, fingolimod can also enhance the endothelial barrier function (Peng et al., 2004; Dudek et al., 2007). However, this effect is likely to be dose-dependent, as higher concentrations or prolonged fingolimod treatment increase endothelial permeability and vascular leakage instead (Shea et al., 2010; Muller et al., 2011). Fingolimod is highly lipophilic and can cross the BBB so it may exert a direct effect on CNS (Fu et al., 2015).

Based on previous studies that fingolimod reduced IR-induced tissue injury in the kidney (Troncoso et al., 2001) and liver (Anselmo et al., 2002), animal models of stroke were performed (Table 1). Most results indicated a beneficial role of fingolimod in tMCAO (Czech et al., 2009; Shichita et al., 2009; Wacker et al., 2009; Hasegawa et al., 2010; Pfeilschifter et al., 2011a,b; Wei et al., 2011; Kraft et al., 2013), thromboembolic stroke model (Campos et al., 2013) and photothrombotic stroke model (Brunkhorst et al., 2013; Li X. et al., 2017; Shang et al., 2020). In contrast, a few negative results were found in a pMCAO model (Liesz et al., 2011) and a large hemispheric stroke model when co-administration with rt-PA (Cai et al., 2013).

**TABLE 1:** Animal studies of S1P modulators in ischemic stroke.

Stroke models	Animal species	Drug name	Drug doses	Drug application	Results	References
TMCAO (90 min)	C57Bl/6 mice (male, 10 weeks old)	FTY720	1 mg/kg	At the onset of ischemia (i.p.)	↓ Infarct volume ↑ neurological function 24 h after reperfusion	Czech et al., 2009
TMCAO (60 min)	C57Bl/6 mice (male, 9–17 weeks old)	FTY720	1 mg/kg	5 min before reperfusion/before reperfusion and every 24 h for 3 days (i.v.)	↓ Infarct volume on day 4	Shichita et al., 2009
TMCAO (60 min)	Swiss-Webster ND4 mice (male, adult)	FTY720	0.24 or 1 mg/kg	48 h before ischemia (i.p.)	1 mg/kg FTY720 or 0.24 mg/kg FTY720 combined with hypoxic preconditioning ↓ Infarct volume ↑ neurological function 24 h after reperfusion	Wacker et al., 2009
TMCAO (120 min)	Sprague–Dawley rats (male)	FTY720, SEW2871, VPC23019	FTY720 (0.25 mg/kg, 1 mg/kg), SEW2871 (5 mg/kg), VPC23019 (0.5 mg/kg)	Immediately after reperfusion (i.p.)	FTY720 and SEW2871 ↓ Infarct volume ↑ neurological function at 24 and 72 h after tMCAO, VPC 20319 abrogated the protective effects of FTY720	Hasegawa et al., 2010
PMCAO	C57BL/6 mice (male, 8–10 weeks old)	FTY720	1 mg/kg	48 h before or 3 h after ischemia (p.o.); 48 h before ischemia (i.p.)	Infarct volume and behavioral dysfunction were not altered 7 days after pMCAO	Liesz et al., 2011
TMCAO (90 min for mice, 120 min for rat), pMCAO	C57BL/6 mice (male), Sprague–Dawley rats (male)	FTY720	0.5 mg/kg, 1 mg/kg, 3 mg/kg	Mice tMCAO: 0.5 mg/kg, 1 mg/kg at reperfusion and at 24 h (i.p.); 3 mg/kg 2 h, 24 h, 48 h after reperfusion (p.o.). Rat tMCAO: 1 mg/kg 30 min after reperfusion (i.p.). Mice pMCAO: 1 mg/kg (i.p.) 2 or 4 h after ischemia	Mice tMCAO: FTY720 (0.5 mg/kg, 1 mg/kg, i.p.) ↓ Infarct volume 48 h after tMCAO, FTY720 (1 mg/kg, i.p.) ↑ neurological function 48 h after tMCAO; FTY720 (3 mg/kg, p.o.) ↓ Infarct volume ↑ neurological function at 14 days. Rat tMCAO: ↓ Infarct volume at 22 h after reperfusion. Mice pMCAO: ↓ Infarct volume 20 h after pMCAO.	Wei et al., 2011
TMCAO (90 min or 3 h)	C57BL/6 mice (male, 10 weeks old)	FTY720	1 mg/kg	2 h after onset of ischemia (i.p.)	↓ Infarct volume (tMCAO 3 h, 90 min) and ↑ neurological function (tMCAO 3 h) at 24 h after the induction of ischemia	Pfeilschifter et al., 2011a
TMCAO (120 min)	C57BL/6J mice, *SphK1^–/–^* and *SphK2^–/–^*mice (10–12 weeks old)	FTY720	1 mg/kg	At the onset of ischemia (i.p.)	FTY720 ↓ Infarct volume ↑ neurological function after 24 h. The protective effect was not shown in *SphK2 ^–/–^*mice	Pfeilschifter et al., 2011b
Photothrombosis (PT) (15 min)	C57BL/6 mice (male, 6–12 weeks old)	FTY720	1 mg/kg	Beginning 3 days after PT, for 5 days b.i.d. (i.p.)	↑ Neurological function over 31 days, ↓ reactive astrogliosis ↑ synapse size at day 7	Brunkhorst et al., 2013
TMCAO (3 h)	C57Bl/6 mice (10–12 weeks old)	FTY720, rt-PA	FTY720 (1 mg/kg), rt-PA (10 mg/kg)	Rt-PA (i.v.), FTY720 (i.p) at the end of the tMCAO period.	FTY720 with rt-PA did not alter mortality rate or neurological function at 24 h after the onset of ischemia	Cai et al., 2013
Thromboembolic occlusion	C57BL/6 mice (male)	FTY720, tPA	FTY720 (0.5 mg/kg), tPA (10 mg/kg)	FTY720 (i.p.) 45 min,24 h, 48 h after occlusion; FTY720 + early tPA: tPA 30 min (i.v.) after thrombin injection + FTY720 (i.p.) 30 min, 24 h, 48 h after occlusion; FTY720 + delayed tPA: tPA 3 h (i.v.) after thrombin injection + FTY720 (i.p.) 3 h, 24 h, 48 h after occlusion;	FTY720 or early tPA: ↓ Infarct volume ↑ neurological function 3 days after occlusion; FTY720 + early tPA: further ↑ neurological function; FTY720 + late tPA: ↓ Infarct volume ↑ neurological function 3 days after occlusion; FTY720 ↓ hemorrhagic transformation associated with late tPA.	Campos et al., 2013
TMCAO (2 h)	Sprague–Dawley rats (male)	FTY720	0.25 mg/kg	Immediately after reperfusion (i.p.)	↓ Infarct volume ↑ neurological function 24 h after tMCAO	Hasegawa et al., 2013
TMCAO (60 min or 90 min)	C57Bl/6 mice, *Rag1^–/–^* mice (male, 6–8 weeks old)	FTY720	1 mg/kg	Immediately before reperfusion (i.p.)	↓ Infarct volume on day 1 and day 3, ↑ neurological function on day 1 after 60-min tMCAO in wild-type mice but not in lymphocyte-deficient *Rag1^–/–^* mice after 90-min tMCAO	Kraft et al., 2013
TMCAO (60 or 90 min)	ICR mice (male, 7 weeks old)	S1P, FTY720	S1P (1 nmol/0.5 μL) FTY720 (3 mg/kg)	FTY720 (i.p.) immediately after reperfusion; S1P + FTY720: microinjection of S1P into the corpus callosum 24 h before tMCAO, FTY720 (i.p.) 30 min before S1P microinjection or tMCAO	FTY720 ↓ Infarct volume ↑ neurological function 22 h after reperfusion of 90-min tMCAO. S1P ↑ Infarct volume ↓ neurological function 22 h after reperfusion of 60-min tMCAO, FTY720 attenuate the augmented damage caused by S1P.	Moon et al., 2015
TMCAO (45 min)	C57BL/6J mice (male, adult)	LASW1238, FTY720	LASW1238 (3 mg/kg, 10 mg/kg), FTY720 (1 mg/kg)	Immediately after reperfusion (i.p.)	LASW1238 (10 mg/kg) ↓ Infarct volume at 24 h after reperfusion	Brait et al., 2016
TMCAO (1 h)	Sprague–Dawley rats (male)	FTY720	0.5 mg/kg	24 h before surgery, and continued every other day (i.p.)	↓ Infarct volume and ↓ memory deficit on day 7 after the surgery	Nazari et al., 2016
PT (20 min)	C57/B6 mice (male, adult)	FTY720	0.5, 1, or 2 mg/kg	2 h after ischemia induction and continued every day (i.p.)	↓ Infarct volume at day 1 and 3 after PT, ↑neurological function at day 1,3,5,7 after PT	Li X. et al., 2017
TMCAO (45 min)	C57BL/6 and lymphocyte-deficient *Rag2^–/–^* mice (male, 12–16 weeks old)	FTY720	1 mg/kg	Immediately after reperfusion (i.p.)	↓ The degree of hemorrhagic transformation in *Rag2^–/–^* mice at 48 h of reperfusion	Salas-Perdomo et al., 2019
TMCAO (90 min)	Sprague–Dawley rats (male)	FTY720	0.5, 1, and 2 mg/kg	Immediately at 1 h after reperfusion (i.p.)	FTY720 (2 mg/kg) ↓ infarct volume ↑ neurological function 24 h after reperfusion	Ji et al., 2019
PT (15 min)	C57BL/6 mice (male)	FTY720	0.3 mg/kg	First dose given 24 h post-stroke, then for 1, 7, 14 consecutive days (i.p.)	↑ Neurological function at day 7 after PT, ↑ angiogenesis in the ischemic boundary at day 14	Shang et al., 2020
TMCAO (60 min)	Wistar rats and CD-1 mice	Probucol (inhibitor of S1P transporter)	30, 60 mg/kg	Immediately after tMCAO (i.p.)	↓ Infarct area after 24 h of reperfusion	Nakagawa and Aruga, 2020
TMCAO (60 min), pMCAO	C57BL/6J mice (adult)	CYM-5442	3 mg/kg	0–6 h after occlusion (pMCAO); immediately before reperfusion (tMCAO)	↓ Infarct size 24 h after pMCAO, ↓ Infarct size 24 h after tMACO.	Nitzsche et al., 2021

*TMCAO, transient middle cerebral artery occlusion; pMCAO, permanent occlusion of the middle cerebral artery; PT, photothrombosis; i.p., intraperitoneally; i.v., intravenously; p.o., by oral gavage; h, hours; min, minutes.*

Encouraged by favorable preclinical data, clinical trials on fingolimod in patients with ischemic stroke have also been conducted. In an open-label pilot trial of patients presented beyond the 4.5 h time window for thrombolytic therapy with anterior circulation infarction, oral fingolimod was given 0.5 mg daily for 3 consecutive days (Fu et al., 2014). Results showed that from baseline to 7 days, enlargement of infarct volume was more restricted and microvascular permeability was reduced in patients who received fingolimod plus standard treatment than in patients receiving standard treatment alone (Fu et al., 2014). Fingolimod treatment was also associated with better neurological function recovery (Fu et al., 2014). In a multi-center trial of patients with anterior or middle cerebral artery occlusion, alteplase plus oral fingolimod 0.5 mg daily for 3 consecutive days were given (Zhu et al., 2015). Patients who received the combination therapy had smaller lesion volume at day 1 and better clinical outcomes at day 90 than patients who received solely alteplase (Zhu et al., 2015). In extended time windows from 4.5–6 h post stroke, patients receiving fingolimod along with alteplase also had a favorable shift of 90-day modified Rankin Scale score (Tian et al., 2018). No adverse effect was reported in the clinical trials mentioned above. More studies aiming to determine whether fingolimod enhances the action of endovascular treatment (NCT04629872) and a combination of fingolimod with alteplase in conjunction with thrombectomy (NCT04675762) are underway. Although the available clinical data are promising, it is noteworthy that current trials were not double-blinded and the number of participants was small and included mainly Asian patients. Therefore, it is still too early to confirm the role of fingolimod in the treatment of ischemic stroke. Additional large-scale, well-designed experiments are warranted.

### Other S1PR Modulators

As potential substitutes to fingolimod, other S1PR modulators with better specificity, improved pharmacokinetic properties are under study. SEW2871 and LASW1238 are selective agonists of S1PR1 and were shown to reduce infarct volume after tMCAO (Hasegawa et al., 2010; Brait et al., 2016). CYM-5442 is another S1PR1-selective agonist reported to have preferential distribution to the brain after systemic administration (Gonzalez-Cabrera et al., 2012). Besides, CYM-5442 is shown to induce a shorter duration of lymphopenia than fingolimod (Gonzalez-Cabrera et al., 2012). In a recent experiment of a pMCAO model, CYM-5442 reduced 24-hour infarct size when administered 0-6 hours after occlusion and the beneficial effect was demonstrated to depend on endothelial S1PR1. In contrast, in another tMCAO model, siponimod, a S1PR modulator of S1PR1 and S1PR5, did not reduce stroke size in middle-aged mice despite significant lymphopenia. More basic and clinical researches are in progress to dig deeper into the therapeutic potential of other S1PR modulators for ischemic stroke.

## Future Therapeutic Perspectives

### The Underlying Mechanisms of S1P Signaling in Ischemic Stroke Need Further Study

S1P modulators have shown promise in experimental models and some small-scale clinical trials of stroke. Yet their mechanisms of action are not fully understood. Optimal targeting strategies remain to be defined. A recent study revealed the pivotal role of endothelial S1P signaling in supporting BBB and maintaining perfusion in the penumbra area of ischemic stroke (Nitzsche et al., 2021). This argues against the application of functional S1PR1 antagonists, which may also block endothelial S1P signaling. Since most S1PR1 agonists also induce lymphopenia (Brait et al., 2016; Nitzsche et al., 2021), joint targeting of lymphocyte and endothelial cell receptors with S1PR1 agonists is suggested. Future application of S1P modulation in ischemic stroke treatment depends on an in-depth understanding of the mechanism of action, and on the ability to have a fine temporal and spatial regulation of the signal pathway. With more studies being undertaken, strategies for developing new-generation drugs with superior attributes will be provided.

### To Develop More Specific S1PR Modulators and Seek Other Targets in S1P Signaling Pathway in Ischemic Stroke Treatment

Fingolimod, the S1PR modulator used in most preclinical and clinical studies so far, is non-specific and desensitizes brain endothelial S1PR1 at high doses as discussed above. Moreover, it induces long-lasting lymphopenia (Gonzalez-Cabrera et al., 2012). As the contribution of different S1PR on ischemic stroke is not fully elucidated (Brait et al., 2016), drugs with more specificity could be not only safer but also more efficacious. Besides S1PR, S1P signaling is providing more therapeutic targets such as S1P transporters and metabolic enzymes. New biased S1PR agonists have been developed that do not cause receptor desensitization and do not induce lymphopenia (Poirier et al., 2020). ApoM-Fc is a soluble carrier for S1P, which can specifically activate endothelial S1P receptors but do not influence circulating lymphocyte numbers (Swendeman et al., 2017). With genetic approaches being applied, the roles of SphK and other enzymes in S1P signaling shall be further elucidated (Gaire and Choi, 2021). New pharmacological advances can be expected.

### Timing, Drug Formulation Shall Be Considered and Catering to the Therapeutic Strategy of Ischemic Stroke

The appropriate timing for drug administration is vital for ischemic stroke treatment, especially when considering the narrow time window for thrombolytic therapy and dramatically different series of events occurring at different time points after stroke. Medications for stroke treatment therefore shall be fast-acting. As for fingolimod, lymphopenia is induced within 6 h of the first dose and the lymphocyte count readily returns to baseline within 72 h of the last dose (Fu et al., 2015). Treatment duration is another problem in need of attention. A study showed that S1P1 agonist LASW1238 reduced infarct size in a tMCAO mouse model only when the lymphopenia state was induced for 24 hours. Defining the best dose and duration of drugs to ensure sufficient but not excessive lymphopenia is thus critical (Brait et al., 2016). Besides, the optimal route of drug delivery should also be considered. In cases of severe stroke, intravenous administration is preferable to oral administration for the impaired ability of swallowing and absorption in these patients (Fu et al., 2015).

### To Reveal the Role of S1P Signaling in Functional Recovery and Post-stroke Sequelae

As mentioned above, the role of S1P signaling in neurogenesis, synaptogenesis, angiogenesis, white matter remodeling, and other processes of functional recovery after stroke is poorly studied. Besides, due to a reduced mortality of ischemic stroke, stroke prevalence is on the rise. Other than sensorimotor deficits, neuropsychiatric sequelae such as depression dramatically reduce the quality of life. In light of this, assessing the role of S1P signaling in post-stroke sequelae is promising.

### Methodological Quality of Preclinical Trials Shall Be Improved and Close Cooperation Between Preclinical and Clinical Studies Are Called For

Although there are dozens of preclinical trials on S1P modulators in ischemic stroke treatment as described above, it should be noted that these experiments are heterogeneous in design. The ischemic stroke models, occlusion times, doses of drug, timing, and duration of treatment, route of administration are different (Table 1). Methodological problems might limit the effective translation from bench to bedside. In light of this, preclinical studies should be better designed to incorporate sex, age, and comorbidity factors. On the other hand, problems encountered in clinical trials can be brought back to mechanism research. The close cooperation between preclinical and clinical studies would be valuable to bring new insight into S1P signaling modulation in ischemic stroke treatment.

## Conclusion

Numerous preclinical and clinical studies suggest that S1P signaling pathway is actively engaged in ischemic stroke pathology. Several small-scale clinical trials investigating the effect of fingolimod in ischemic stroke patients have shown promising results. Other S1PR modulators with better specificity and pharmacokinetic property are under development. Synergistic interaction between preclinical and clinical studies would help to achieve new pharmacological advances in ischemia stroke treatment.

## Author Contributions

S-QZ drafted the initial version of the manuscript. JX, MC, L-QZ, and KS collected information and edited the manuscript. CQ and D-ST directed the work, reviewed and edited the manuscript. All authors contributed to the article and approved it for publication.

## Conflict of Interest

The authors declare that the research was conducted in the absence of any commercial or financial relationships that could be construed as a potential conflict of interest.

## Publisher’s Note

All claims expressed in this article are solely those of the authors and do not necessarily represent those of their affiliated organizations, or those of the publisher, the editors and the reviewers. Any product that may be evaluated in this article, or claim that may be made by its manufacturer, is not guaranteed or endorsed by the publisher.
